# 

**Published:** 1933

**Authors:** 


					CONTENTS.
Page
Editorial Note
149
The History of the Bristol Medico - Chirurgical Journal.
By Patrick Watson-Williams, M.D.
151
' Bristol's Contributions to Medical Progress
- :
165
I
The Centenary of the Foundation of the Bristol Medical
School
183
Schola Medicinae Bristol (Review) ., .. .. ?
199

				

